# Influence of Prior Influenza Vaccination on Antibody and B-Cell Responses

**DOI:** 10.1371/journal.pone.0002975

**Published:** 2008-08-20

**Authors:** Sanae Sasaki, Xiao-Song He, Tyson H. Holmes, Cornelia L. Dekker, George W. Kemble, Ann. M. Arvin, Harry B. Greenberg

**Affiliations:** 1 Department of Medicine, Stanford University School of Medicine, Stanford, California, United States of America; 2 Department of Microbiology and Immunology, Stanford University School of Medicine, Stanford, California, United States of America; 3 Veterans Affairs Palo Alto Health Care System, Palo Alto, California, United States of America; 4 Department of Health Research and Policy, Division of Biostatistics, Stanford University School of Medicine, Stanford, California, United States of America; 5 Department of Pediatrics, Stanford University School of Medicine, Stanford, California, United States of America; 6 MedImmune Vaccines, Inc., Mountain View, California, United States of America; University of California San Francisco, United States of America

## Abstract

Currently two vaccines, trivalent inactivated influenza vaccine (TIV) and live attenuated influenza vaccine (LAIV), are licensed in the USA. Despite previous studies on immune responses induced by these two vaccines, a comparative study of the influence of prior influenza vaccination on serum antibody and B-cell responses to new LAIV or TIV vaccination has not been reported. During the 2005/6 influenza season, we quantified the serum antibody and B-cell responses to LAIV or TIV in adults with differing influenza vaccination histories in the prior year: LAIV, TIV, or neither. Blood samples were collected on days 0, 7–9 and 21–35 after immunization and used for serum HAI assay and B-cell assays. Total and influenza-specific circulating IgG and IgA antibody secreting cells (ASC) in PBMC were detected by direct ELISPOT assay. Memory B cells were also tested by ELISPOT after polyclonal stimulation of PBMC in vitro. Serum antibody, effector, and memory B-cell responses were greater in TIV recipients than LAIV recipients. Prior year TIV recipients had significantly higher baseline HAI titers, but lower HAI response after vaccination with either TIV or LAIV, and lower IgA ASC response after vaccination with TIV than prior year LAIV or no vaccination recipients. Lower levels of baseline HAI titer were associated with a greater fold-increase of HAI titer and ASC number after vaccination, which also differed by type of vaccine. Our findings suggest that the type of vaccine received in the prior year affects the serum antibody and the B-cell responses to subsequent vaccination. In particular, prior year TIV vaccination is associated with sustained higher HAI titer one year later but lower antibody response to new LAIV or TIV vaccination, and a lower effector B-cell response to new TIV but not LAIV vaccination.

## Introduction

Influenza virus can cause severe respiratory illness in young children and adults, which can lead to death, especially in the elderly over 65 years old [1]. On average 5–20% of the population is infected with influenza virus in the United States every year [2], [3], resulting in a considerable economic burden [4]. To prevent seasonal influenza, the Centers for Disease Control and Prevention recommends annual influenza vaccination. Humoral antibody to the influenza hemagglutinin (HA), which is induced by natural infection or vaccination, has an important role in protection. Most adults and older children have pre-existing levels of antibody because of prior infection or vaccination [5], [6]. However, antigenic drift of influenza virus, which is caused by an accumulation of point mutations in the HA and neuraminidase (NA) genes, occurs both in influenza A and B viruses [7]–[9]. An individual who was infected by or previously vaccinated against influenza viruses circulating in prior years may still be susceptible to a new virus strain. Therefore, influenza vaccines are reformulated each year based on the results of international surveillance that predict the virus strains that will circulate in a subsequent year.

There are two types of vaccines available in the US: inactivated trivalent influenza vaccine (TIV) and live attenuated influenza vaccine (LAIV). Both vaccines contain two influenza A viruses (H1N1 and H3N2) and one influenza B virus. Inactivated influenza vaccine has been used since 1945 and the accumulated studies confirm that the hemagglutination inhibition (HAI) antibody response after vaccination correlates with protection against subsequent influenza infection [10]–[12]. Therefore, serum antibody responses are used as a surrogate marker for the efficacy of TIV for the purpose of annual re-licensing. LAIV was licensed in the US in 2003. Intranasal administration of LAIV induced a robust serum and mucosal antibody response in young, non-immune children [13], [14]. However, the magnitude of influenza-specific antibody response in the serum of older children and adults is generally substantially lower compared to what is seen following immunization with TIV [5], [15], [16]. Despite the lower serum response observed in the circulation following LAIV immunization in healthy adults and older children, vaccine efficacy appears to be at least comparable to TIV [17]–[19]. TIV is approved for use in people older than 6 months, including healthy people and people with chronic medical conditions [20]. LAIV is approved for use in healthy people 2–49 years of age who are not pregnant and are without a history of asthma or recurrent wheezing [20]. Therefore, most healthy adults under 49 years of age and healthy children older than 2 years of age can choose between these two vaccines in the US.

Although annual vaccination is recommended for all ages, the efficacy of repeated vaccination with TIV has been debated for many years [21]–[23]. The Hoskins study concluded that repeated vaccination was not protective for the long term, while more recent studies have generally, but not universally, concluded that repeated vaccination was effective over the long term [22], [24], [25]. Many factors, including type of vaccine (live or inactivated), match of antigenicity between vaccine and circulating influenza strains, age, and study design, influenced the evaluation of vaccine efficacy [6], [23], [26]. While several studies have shown that TIV vaccination resulted in higher serum antibody titers after one year compared to unvaccinated controls [21], [27]–[30], other immune markers, such as memory B-cells and long-lived plasma cells have not been thoroughly investigated. In our previous study during the 2004–2005 influenza season, we observed that the titer of neutralizing antibody to H3N2 in serum was significantly higher in adult subjects who received TIV during the previous 2003–2004 season compared to those who had LAIV [5]. On the other hand, a year after vaccination, the levels of influenza-specific circulating memory B cells were similar between LAIV and TIV recipients [5]. However, the influences of prior year vaccination on the antibody and B-cell responses to re-vaccination have not been analyzed in detail, especially after LAIV vaccination. To address this issue in a new study carried out during the 2005–2006 influenza season, we investigated the serum antibody response, effector B-cell (antibody secreting cell, ASC) response, and memory B-cell response in adult subjects who were not or were vaccinated with either LAIV or TIV in the prior year, and received a new dose of the influenza vaccine (either TIV or LAIV).

## Materials and Methods

### Human subjects and vaccination protocols

Sixty one healthy adults (age range 22–49 years) were enrolled in this study between September and early December 2005. These subjects were randomized at a 1∶1 ratio to receive one dose of either TIV (Fluzone®, Aventis-Pasteur) or LAIV (FluMist®, MedImmune). In the LAIV group (n = 31), 14 subjects did not receive influenza vaccine in the prior year, 4 received LAIV, and 13 received TIV. In the TIV group (n = 30), 14 subjects did not receive influenza vaccine in the prior year, 9 received LAIV, and 7 received TIV. None of the subjects had chronic cardiovascular or pulmonary disorders, a history of asthma or reactive airway disease, autoimmune disease, immunosuppression, immunodeficiency or impaired immunologic functions, allergies to any component of the vaccine including thimerosal, or allergy to eggs or egg products. The study protocol was approved by the institutional review board at Stanford University. Written informed consent was obtained from all participants.

Three blood samples were obtained from each subject: on day 0 prior to vaccination, and day 7 (range 7–9) and 30 (range 21–35) after vaccination. The 2005–2006 TIV and LAIV vaccines both contained the A/New Caledonia/20/99 (H1N1) and B/Jiangsu/10/2003 (B) strains. The third strain was A/California/7/2004 (H3N2) in LAIV and A/NewYork/55/2004 (H3N2, an A/California/7/2004-like strain) in TIV. The prior year 2004–2005 TIV and LAIV vaccines both contained A/New Caledonia/20/99 (H1N1) and A/Wyoming/03/2003 (H3N2) strains. The third strain was B/Jiangsu/10/2003 in TIV and B/Jilin/20/2003 in LAIV.

### 2-color ELISPOT assay for antibody secreting cells (ASC)

PBMC were isolated from heparinized whole blood by ficoll gradient centrifugation and resuspended in RPMI1640 (Gibco, Grand Island, NY) containing 10% fetal calf serum (FCS, Hyclone, South Logan, UT). Total and influenza-specific IgA and IgG ASC were detected simultaneously as previously described [5]. In brief, 96-well plates (Immobilon P membrane, MAIPN4510, Milipore, Billerica, MA) were coated with TIV at a total concentration of 9 µg/ml in PBS to detect influenza-specific ASC, or coated with affinity purified goat anti-human IgA+IgG+IgM (H+L) (KPL, Gaithersburg, MD) in PBS to detect total ASC. The influenza coating antigen was a 1∶1 mixture of TIV 2002–2003, which had the same components as TIV 2003–2004, and TIV 2005–2006. Wells coated with PBS served as negative controls. Plates were incubated overnight at 4°C and then blocked for 2 hr at 37°C with RPMI 1640 containing 10% FCS, 100 units/ml of penicillin G and 100 µg/ml of streptomycin (Gibco), referred to as complete medium. Freshly isolated PBMC or cultured PBMC (see below) were resuspended in complete medium containing peroxidase conjugated goat anti-human IgA antibody (Sigma, St. Louis, MO) and phosphatase conjugated goat anti-human IgG (H+L) antibody (KPL), dispensed into ELISPOT plates and incubated for 4 hrs or longer at 37°C in a CO_2_ incubator. Plates were washed with PBS and developed first with an AEC substrate kit for peroxidase (Vector, Burlingame, CA) and subsequently with a Blue alkaline phosphatase substrate kit (Vector). IgA ASC was visualized as red spots and IgG ASC as blue spots in the same wells. The quantity of ASC per well was determined by counting the spots with an ELISPOT plate reader (CTL-ImmunoSpot® S5 Macro Analyzer, with the software ImmunoSpot version 3.2, Cellular Technology Ltd. OH). Nonspecific spots detected in the negative control (PBS) wells were subtracted from the counts of influenza-specific and total ASC.

### Memory B-cell assay

To detect memory B cells, PBMC were stimulated to induce differentiation of memory B-cells into ASC, as described by Crotty et al. [31]. PBMC were distributed into 24-well plates (Costar, Corning, NY) at 5×10^5^ cells/well in complete medium with 55 µM of 2-mercaptoethanol and a mixture of polyclonal mitogens: 1/100,000 dilution of pokeweed mitogen extract (PWM, kindly provided by Dr. R. Ahmed, Emory University, Atlanta, GA), 6 µg/ml of CpG olygonucletide ODN-2006 (Invivogen, San Diego, CA), and 1/10,000 dilution of fixed *Staphylococcus aureus* Cowan (SAC) (Sigma). Duplicate wells were cultured for each subject. Cells cultured in complete medium with 2-mercaptoethanol but without CpG, SAC, and PWM served as negative controls. Cells were cultured for 5–6 days at 37°C in a CO_2_ incubator. The cultured cells were washed twice with complete medium and transferred into coated ELISPOT plates as described above. To adjust for the background of the ELISPOT assay, we subtracted the number of nonspecific spots (PBS control) from the count of influenza-specific or total ASC. We then estimated the number of ASC differentiated from memory B cells by subtracting the number of influenza-specific or total ASC in unstimulated samples (negative controls) from those in stimulated samples. Data are presented as the percentage of influenza–specific ASC per total ASC per culture well [31].

### Serum hemagglutination inhibition assays

Serum samples were pretreated with receptor-destroying enzyme (RDE) (Denka Seiken, Tokyo) overnight at 37°C and subsequently heated at 56°C for 45 min. Hemagglutination inhibition assay (HAI) was performed as previously described [32]. In brief, serially diluted 25 µl aliquots of serum samples in PBS were mixed with 25 µl aliquots of virus, corresponding to four HA units, in V-bottom 96-well plates (Nunc, Rochester, NY) and incubated for 30 min at room temperature. At the end of the incubation, 50 µl of 0.5% chicken (for influenza H1) or turkey (for influenza H3 viruses) red blood cells were added and incubated for a minimum of 30 min. The serum HAI antibody titer of a given sample was defined as the reciprocal of the last serum dilution with no hemagglutination. A titer of 2 was assigned to all samples in which the first dilution (1∶4) was hemagglutination-negative.

### Statistical analysis

Throughout, data are summarized by group as the mean±1 standard error when skewness of data is less than 1. For skewness >1, data are log transformed and summarized by group as the geometric mean (and 95% confidence interval). Two-sample comparisons employed unpaired t-tests for normally distributed data (with Welch's correction if sample variances were sufficiently different). We employed The Wilcoxon signed rank test for two related samples (the data before and after vaccination). Correlation was assessed using Spearman's coefficient to include detection of any monotonic association.

We employed generalized estimating equations (GEE) for regression analysis of longitudinal data [33]. GEE analysis assumed the variance is a quadratic function of the mean under a logarithm link function. The GEE regression model assumed that ASC response is a function of vaccine type, linear and quadratic terms for days after vaccination, year, and interactions of vaccine type with linear and quadratic terms for days after vaccination. Because the sampling of days differed between years, the model did not include a vaccine type by year interaction term, thereby assuming differences between TIV and LAIV did not depend on year (2004 vs. 2005). The primary comparison of interest was the difference between TIV and LAIV in fitted means between days 7 and 11 post-vaccination. Attained significance levels of P<0.05 are considered to be statistically significant.

Adjustments for multiple comparisons were made as indicated, via a sequential Bonferroni procedure [34]. Analyses were conducted in Graph pad Prism v. 5.0 (GraphPad Software, Inc., San Diego, CA), SAS version 9.1 (SAS Institute, Cary, NC), and S-Plus (Insightful Corp., Seattle, WA). All SAS and S-Plus code is available upon request.

## Results

### Influence of TIV or LAIV vaccination in the prior year on the serum antibody responses to new vaccination

**Figure 1:**
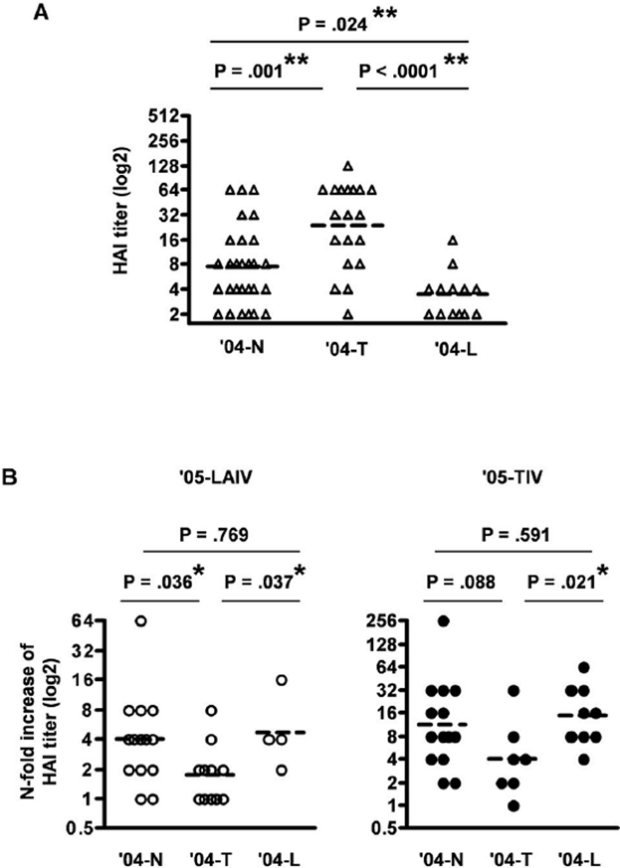
HAI titer to influenza A/California (H3N2) strain before and 30 days after TIV or LAIV administration. Subjects are divided into three groups based on the status of influenza vaccination in the prior year (2004–2005): TIV ('04-T), LAIV ('04-L), or no vaccination ('04-N). A: Baseline HAI titer. B: fold-increase of HAI titer from baseline to day 30 of LAIV ('05-LAIV) or TIV ('05-TIV). The dashed-bars in each graph indicate the geometric mean values. **, significant difference after sequential Bonferroni adjustment for multiple comparisons. *, results with a P value of <.05 in an individual unpaired t-test but nonsignificant after sequential Bonferroni adjustment for multiple comparisons.

To investigate the influence of prior year vaccination on the serum antibody response to new vaccination, we compared the HAI response between the three sub-groups of subjects who either received TIV or LAIV influenza vaccines or were not vaccinated in the prior year. We denote these prior year recipient groups of TIV, LAIV or no vaccine as the ′04-T, ′04-L, and ′04-N groups with the following enrollments: ′04-T group, n = 19, ′04-L group, n = 13, or ′04-N group, n = 28. We focused on HAI titer against the H3N2 subtype virus because influenza A/H3N2 has, in recent years, generally been a more virulent strain compared to the other strains and has exhibited more antigenic variation from season to season. H3N2 subtype viruses predominated in the USA during the 2004–2005 influenza season and the strain has been drifting over the past few years. Among these three treatment sub-groups, we observed a significant difference in HAI titer against the H3N2 strain at baseline (day 0), prior to immunization in the current study (Figure 1A). In agreement with previous studies by our group and others [5], [21], [30], the ′04-T group had a significantly higher baseline HAI titer ([24: 14–42], geometric mean with 95% confidence interval) compared to the ′04-N group ([7: 5–11], P = .001, unpaired t-test) and the ′04-L group ([3: 2–5], P<.0001, unpaired t-test with Welch's correction). Although our previous report showed that both TIV and LAIV induced increased serum antibody titers one month after vaccination [5], a year later, the ′04-L group had a significantly lower geometric mean HAI titer compared to the ′04-N group (P = .024, unpaired t-test).

One month after vaccination, both LAIV (′05-LAIV) and TIV (′05-TIV) induced significant increases in geometric mean HAI titer against H3N2 compared to baseline (P<.0001, Wilcoxon signed rank test), and ′05-TIV recipients reached significantly higher geometric mean HAI titer compared to ′05-LAIV recipients ([79: 55–114] vs. [28: 19–42], P = .0003, unpaired t-test). These data suggest that the vaccines used in this study induced similar antibody responses as described in previous studies [5].

After ′05-LAIV vaccination, the mean fold-increase in HAI titer in the ′04-T group [2: 1–3] was smaller than either the ′04-N group or ′04-L group ([4: 2–7] and [5: 1–19], P = .036 and .037, respectively, unpaired t-test) (Figure 1B). This same tendency was observed in ′05-TIV recipients, in which there was a smaller fold-increase of HAI titer in the ′04-T group than the ′04-L group (P = .021, unpaired t-test) (Figure 1B). Taken together, these results suggest that the status of prior year influenza vaccination influences the influenza strain-specific antibody level a year after vaccination as well as the changes in antibody titer after new annual flu vaccination. Compared to subjects who were not vaccinated, TIV vaccination in the prior year resulted in higher baseline serum antibody, while LAIV vaccination resulted in lower serum antibody titers one year later. One month after vaccination with either ′05-LAIV or ′05-TIV, the fold-increase in HAI titers was lower in the subjects who received TIV in the prior year than in those who received LAIV previously.

### Influence of TIV or LAIV immunization in the prior year on the effector B-cell responses to new vaccination

**Figure 2:**
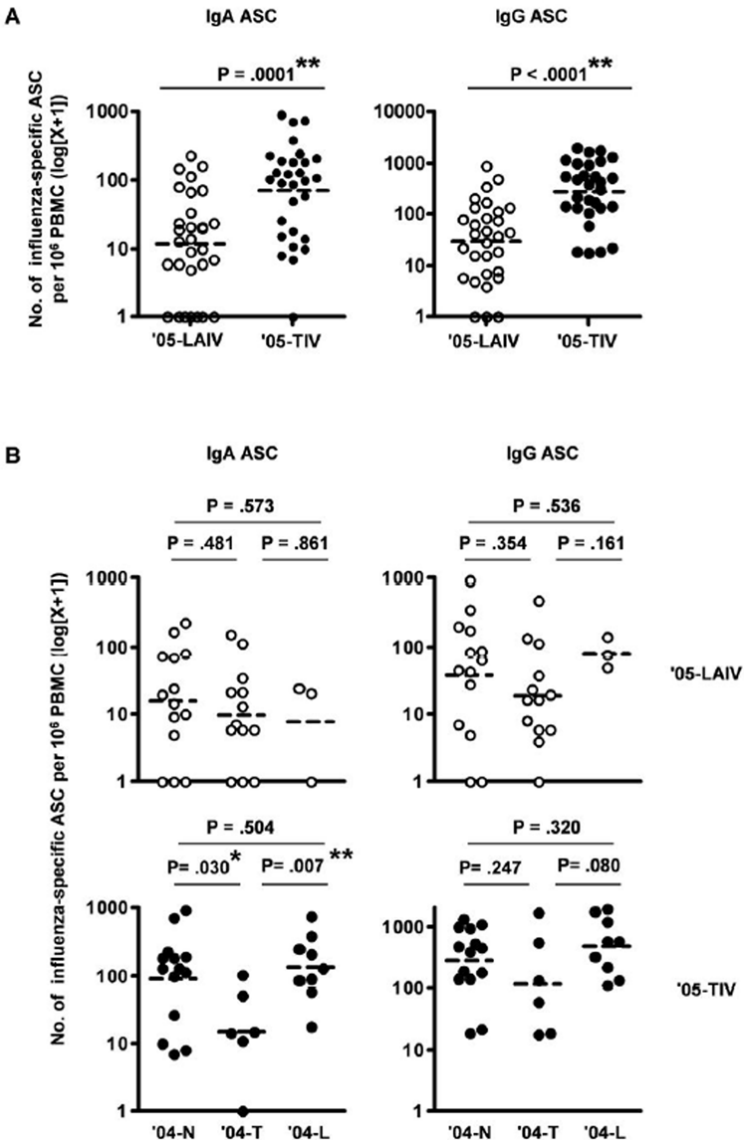
Frequency of circulating influenza-specific ASC after LAIV or TIV vaccination. Influenza-specific IgA ASC and IgG ASC were measured with a direct ELISPOT assay at days 7–9 after LAIV ('05-LAIV) or TIV ('05-TIV) vaccination. A: Influenza-specific IgA and IgG ASC in all subjects after administration of '05-LAIV or '05-TIV. B: Influenza-specific IgA and IgG ASC after '05-LAIV or '05-TIV in the three sub-groups based on influenza vaccination status in the prior year: TIV ('04-T), LAIV ('04-L), or no vaccination ('04-N). The dashed-bars in each graph indicate the geometric mean values. **, P<.01 unpaired t-test (A) or significant difference after adjustment for multiple comparisons (B). *, result with a P value of <.05 in an individual test but not significant after adjustment for multiple comparisons (B).

Before vaccination (day 0), influenza-specific IgA and IgG ASC were not detected in the circulation of most adults (only 3 out of 31 adults vaccinated in the prior year and only 2 out of 27 adults not vaccinated had detectable ASC with one ASC/million PBMC, all others were below the detection threshold). The frequency of influenza-specific ASC in PBMC obtained 7–9 days after ′05-LAIV or ′05-TIV vaccination was determined by direct ELISPOT assay. In agreement with previous findings [5], the mean number of effector IgA and IgG ASC per million PBMC was significantly greater in ′05-TIV recipients than in ′05-LAIV recipients (IgA ASC: [70: 37–130] vs. [11: 5–21], IgG ASC: [275: 160–472] vs. [29: 14–58], P = .0001 and <.0001, respectively, unpaired t-test) (Figure 2A). Next, we compared the effector B-cell responses to ′05-LAIV or ′05-TIV in recipients who had received LAIV, TIV or no vaccine in the prior year (Figure 2B). Significant differences were not detected in the number of influenza-specific IgA or IgG ASC among the three groups following ′05-LAIV vaccination (P≥.161 for all comparisons, unpaired t-test). In contrast, in ′05-TIV recipients, the mean number of IgA ASC in the ′04-T group [14: 2–79] was lower than that in the ′04-N group [90: 36–220] or ′04-L group [135: 57–315]. These differences are statistically or marginally significant between the ′04-T and ′04-L groups (P = .007, unpaired t-test) and the ′04-T and ′04-N groups (P = .03, unpaired t-test) after adjustment for multiple comparisons (Figure 2B). The effector IgG ASC response after ′05-TIV in the three groups followed a similar trend to the effector IgA ASC responses, although the differences were not significant (Figure 2B). These data suggest that prior year vaccination with TIV, but not LAIV, reduced the IgA effector B-cell response to new TIV immunization and may have a similar effect on the IgG B-cell response.

### Kinetics of influenza-specific effector B-cell responses to TIV and LAIV

**Figure 3:**
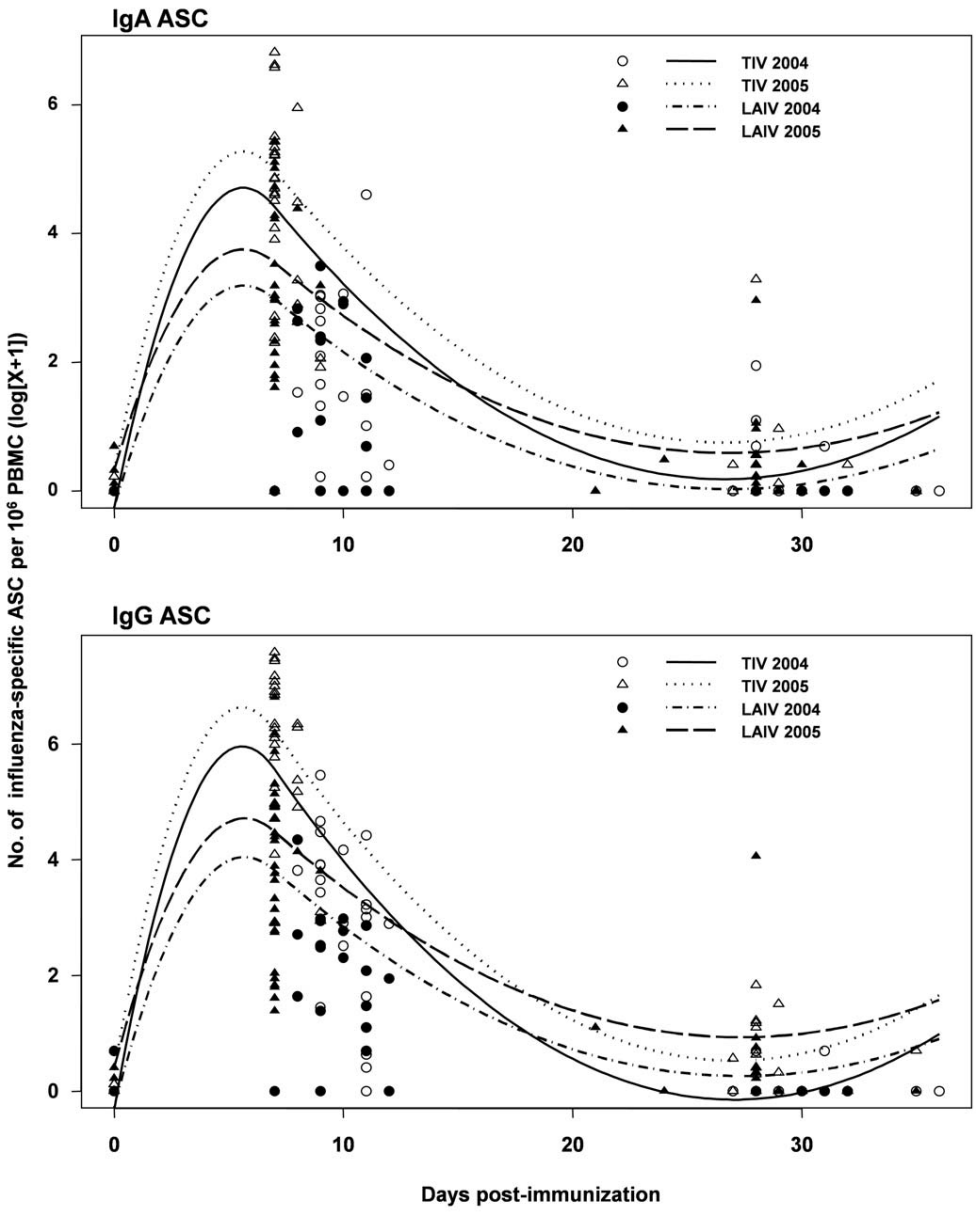
Kinetics of effector IgA and IgG ASC responses after TIV or LAIV vaccination. Plotted is the frequency of influenza-specific effector IgA and IgG ASC as a function of days after TIV or LAIV vaccination for the 2004 and 2005 studies. Observed data are plotted as circles and triangles. Lines provide the fit of the GEE regression model. Of interest was comparing the difference in the slopes of these fitted lines over days 7–11 post-vaccination between TIV and LAIV. Regression analysis assumed that this difference between TIV and LAIV did not depend on year (2004 vs. 2005).

The peripheral blood effector B-cell response after TIV vaccination may be transient [35], [36], while the kinetics of the effector B-cell response after LAIV vaccination are unknown. To compare the kinetics of effector B cells in the periphery after vaccination with LAIV or TIV, we used the effector B-cell ELISPOT data collected in 2004 [5] and 2005. On day 0 (before vaccination), the number of IgG and IgA effector cells was under the limit of detection in almost all subjects. Effector B cells were detected during days 7–11 after vaccination, and largely disappeared by days 21–36 in both TIV and LAIV recipients (Figure 3). In agreement with previous reports [5], both IgG and IgA ASC response declined from day 7 to day 11 after vaccination with either TIV or LAIV. However, the average rate of decline during this period, defined as the difference in the fitted regression means between days 7 and 11, is significantly greater in the TIV groups compared to the LAIV group (P = .0203 and .0036 for IgA and IgG effector B cells, respectively). These results indicate that the circulating effector B-cell response to TIV subsides faster than that to LAIV.

### Influence of prior TIV or LAIV vaccination status on the memory B-cell responses to new vaccination

**Figure 4:**
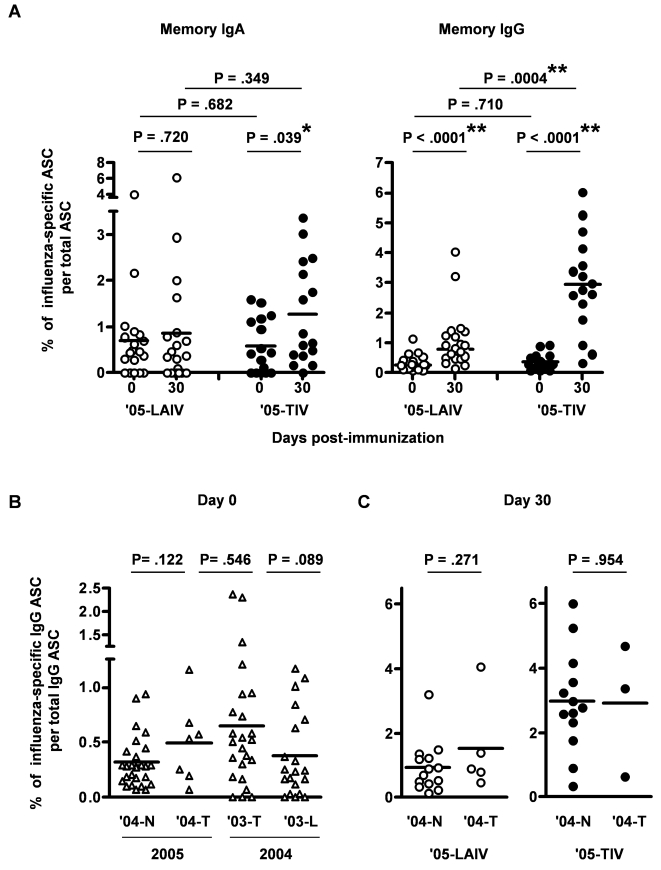
The percentage of circulating influenza-specific memory B cells before and after LAIV or TIV vaccination. Percentages of influenza-specific memory IgG B-cells and IgA B-cells before (day 0) and 30 day (21–35 days in 2005 study, 27–47 days in 2004 study) after LAIV or TIV vaccination were measured. A: Comparisons of the percentages of influenza-specific memory IgA and IgG B-cells on day 0 and day 30 after LAIV ('05-LAIV) or TIV ('05-TIV) vaccination. B: Comparisons of memory IgG B-cell levels before vaccination in sub-groups based on the influenza vaccine received in the prior year: TIV ('03-T, '04-T), LAIV ('03-L), or no vaccination ('04-N). C: Comparisons of memory IgG B-cell levels one month after vaccination with '05-LAIV or '05-TIV between sub-groups '04-N and '04-T. The horizontal bars indicate the mean values. *, P<.05, **, P<.01, unpaired t-test.

Previously we observed that most adults had preexisting influenza-specific memory IgG B cells in their circulation before vaccination [5]. In addition, one month after TIV but not LAIV immunization, the percentage of influenza-specific memory IgG B cells in circulation increased significantly, with a higher percentage of memory IgA and IgG B cells induced by TIV compared to LAIV [5]. In the current study we again studied the memory B-cell levels before and one month after ′05-TIV and ′05-LAIV vaccination in adult subjects (Figure 4). Of the 61 adults, we analyzed the memory B-cell response in the 37 adults who were not involved in the 2004–2005 study. Of these 37 subjects, 8 had received TIV, one had received LAIV, and 28 had not received either vaccine in the prior year. At baseline (day 0), no significant difference was detected in the average percentages of circulating influenza-specific memory IgA and IgG B cells between the ′05-LAIV and ′05-TIV groups (P = .682 and .71, respectively, unpaired t-test) (Figure 4A). One month after immunization, significant increases in the percentage of influenza-specific memory IgG B cells were observed in both ′05-LAIV and ′05-TIV recipients, with the increase in ′05-TIV recipients significantly greater than that in the ′05-LAIV recipients (2.50±0.44, mean±standard error vs. 0.73±0.23, P = .0019, unpaired t-test with Welch's correction). The mean percentage of memory IgA B cells also significantly increased in ′05-TIV recipients (P = .039, unpaired t-test with Welch's correction), while no significant increase was detected in the new ′05-LAIV recipients.

To explore the influence of prior year vaccination on influenza-specific memory B-cell responses, we used the day 0 and day 30 memory B-cell datasets derived from our previous study (2004 study) and the current study (2005 study). These included a ′03-L group and a ′03-T group from the 2004 study, as well as a ′04-N group and a ′04-T group from the 2005 study. A ′04-L group from the 2005 study was not available because only one subject in this study received LAIV in the prior year. We observed a significant increase in mean percentage of influenza-specific memory IgG B cells one month after TIV vaccination in both 2005 and 2004 studies (Figure 4A and [5]), with 69% of the TIV recipients experiencing an increase greater than three-fold. However, in the 2005 study a significant difference was no longer detectable in the baseline levels of memory IgG B cells between the ′04-T group, which received TIV in the prior year, and the ′04-N group, which did not receive an influenza vaccine in the prior year (P = .122, unpaired t-test, Figure 4B). Similarly, although ′05-TIV vaccination in the current study resulted in a greater mean percentage of memory IgG B-cells one month after vaccination than ′05-LAIV (Figure 4A and [5]), in our 2004 study no significant difference was detected in the baseline percentage of the memory IgG B-cells between the ′03-T and ′03-L groups (P = .089, unpaired t-test with Welch's correction) (Figure 4B and [5]). Taken together, these results suggest that regardless of the status of prior year vaccination and the 30 day B-cell memory response following vaccination, the percentage of circulating influenza-specific memory B cells returned to statistically indistinguishable levels one year after vaccination in the adult vaccinees.

One month after either ′05-LAIV or ′05-TIV vaccination, no significant difference was detected in the mean percentage of influenza-specific memory B cells between the ′04-N group and ′04-T group (Figure 4C). In addition, no significant difference was detected in the mean change of percentage influenza-specific memory B cells from baseline between the ′04-N group and ′04-T group (data not shown). Taken together, these results suggest that although TIV and LAIV induced different levels of influenza-specific memory IgG B cells one month after vaccination, prior year vaccination status does not affect memory IgG B-cell responses in the blood one month after vaccination with either TIV or LAIV.

### Correlation between serum antibody and B-cell responses after LAIV or TIV

Most adults have circulating antibodies against influenza viruses due to prior exposure to influenza infection and/or vaccination. To explore the effects of these antibodies on the immune responses induced by a new vaccination, we analyzed the correlations between baseline HAI titer and the following immune responses after vaccination with either ′05-TIV or ′05-LAIV: serological response, defined as the fold-change of HAI from day 0 (baseline) to day 30; and memory B-cell responses, defined as the percentage increase of influenza-specific IgG and IgA memory B cells from day 0 to day 30. For effector B-cell responses, since influenza-specific ASC were not detected at day 0 (Figure 3), we defined effector B-cell response as the number of influenza-specific IgG and IgA ASC per million PBMC at day 7–9. The results of these analyses are summarized in Table 1. A significant inverse correlation was observed between the fold-change of HAI titer after vaccination and baseline HAI titer in both ′05-LAIV and ′05-TIV groups. In the ′05-TIV group, an inverse correlation was also observed between the effector B-cell response and baseline HAI, especially HAI against the H1N1 strain. In the ′05-LAIV group, an inverse correlation was observed between the memory IgA B-cell response and baseline HAI titer against H3N2, but no correlation was observed between memory B-cell responses and the baseline HAI titer in the rest of the combinations. In addition, a significant positive correlation was observed between the serum antibody response and effector B-cell response after vaccination, especially after ′05-LAIV, and between IgG memory B-cell response and serum antibody response in the ′05-TIV group (Table 2).

**Table 1:** Correlation between baseline HAI titer and B-cell immune responses.

Group	Immune responses (change from day 0)	Baseline HAI			
		H3N2		H1N1	
		Spearman's r	P value	Spearman's r	P value
LAIV	HAI (day 30)	**−.5442**	**.0023**	**−.3817**	**.041**
	Effector IgA (day 7)	−.2351	.2195	−.2886	.129
	Effector IgG (day 7)	−.2469	.1966	−.3607	.0545
	Memory IgA (day 30)	**−.6746**	**.0015**	−.2916	.2258
	Memory IgG (day 30)	−.1453	.5529	−.2343	.3344
TIV	HAI (day 30)	**−.7225**	**<.0001**	**−.803**	**<.0001**
	Effector IgA (day 7)	**−.3954**	**.0338**	**−.489**	**.0071**
	Effector IgG (day 7)	−.3076	.1045	**−.3849**	**.0392**
	Memory IgA (day 30)	.1	.723	−.08	.776
	Memory IgG (day 30)	−.2462	.3764	−.4143	.1247

**Table 2:** Relationship between serum HAI and B-cell responses to influenza vaccines

Group	B-cell responses (change from day 0)	Fold-increase HAI (day 30)			
		H3N2		H1N1	
		Spearman's r	P value	Spearman's r	P value
LAIV	Effector IgA (day 7)	**.6621**	**.0001**	**.4592**	**.014**
	Effector IgG (day 7)	**.6553**	**.0002**	**.4849**	**.0089**
	Memory IgA (day 30)	.2245	.3554	−.0267	.9139
	Memory IgG (day 30)	.1784	.465	−.0873	.7224
TIV	Effector IgA (day 7)	.2954	.1198	**.5237**	**.0036**
	Effector IgG (day 7)	.1386	.4735	**.451**	**.0141**
	Memory IgA (day 30)	.1541	.5833	−.0557	.8438
	Memory IgG (day 30)	**.6129**	**.0151**	**.5379**	**.0386**

## Discussion

The goal of this study was to investigate the relationship between prior immune status and B-cell immune responses to influenza vaccination. Here we report that higher levels of preexisting serum HAI antibodies are associated with reduced serum antibody responses after vaccination with either LAIV or TIV. Similar findings have been noted previously [6], [21], [37]–[40]. Moreover, higher levels of preexisting serum antibodies are associated with a reduced peripheral effector B-cell response to TIV but not LAIV. We also found that the serum antibody and effector B-cell responses to vaccination were related to each other. In addition to the similarities between TIV and LAIV, different characteristics regarding their effects on B-cell immune responses to subsequent immunization are also identified such as the kinetics of effector B cells and the influence of preexisting antibodies on the induction of effector B cells.

Influenza A/California, which was a new variant strain of A/Fujian that was dissimilar to the vaccine strain A/Wyoming for the 2004–2005 season and was isolated in our local area, was the dominant circulating H3N2 strain during the 2004–2005 season [2], [3]. The reported efficacy in adults of the influenza vaccines against both circulating influenza A (A/California-like) and B viruses in the 2004–2005 season was 77% for TIV and 57% for LAIV [41]. Half the subjects recruited for the current study conducted during the 2005–2006 season received either TIV or LAIV in the prior year (2004–2005). We observed that subjects who received TIV in the previous year (′04-T group) had significantly higher baseline HAI for H3N2 A/California compared to those who received LAIV (′04-L group) or no vaccination (′04-N group) in the prior year (Figure 1A). Although the A/California used in the HAI assay is antigenically dissimilar to A/Wyoming in the vaccine of the previous year, some antibodies induced by A/Wyoming likely cross-react with A/California [42] and contribute to the protection against the latter [41]. This higher baseline HAI for A/California is likely to reflect the long lasting antibody response induced by TIV in 2004. Although LAIV induced a serum HAI response in the 2004–2005 season [5], the ′04-L group had a lower baseline HAI titer against A/California a year after the previous immunization, even when compared to the ′04-N group. A plausible explanation for the lower baseline antibody level in the ′04-L group might be that subjects in the ′04-N group were exposed to the circulating influenza in the 2004–2005 season and some of them were infected and seroconverted, which resulted in higher antibody levels compared to those in the ′04-L group who were protected from the infection because they received LAIV. In agreement with this explanation, the baseline HAI titer for H1N1 A/New Caledonia, which had not circulated dominantly for several years [2], [3], was comparable between the ′04-N and ′04-T groups (data not shown).

In the ′05-LAIV and ′05-TIV groups, an inverse correlation was observed between baseline antibody and fold-increase of HAI antibody after vaccination (Table 1), suggesting that serum antibody response to vaccination may be affected by the levels of circulating antibodies at the time of vaccination, with a higher baseline antibody level associated with lower immune responses. These results support studies showing weaker serum responses after LAIV in seropositive people compared to the seronegatives [40], [43]. Interestingly, baseline serum HAI antibody titer also affected the peripheral effector B-cell response to ′05-TIV, but not ′05-LAIV which is consistent with prior studies demonstrating that ASC responses are a more sensitive indicator than HAI of vaccine “take” following LAIV administration [5]. The route of administration and type of vaccine can influence the induction of effector B-cell response. Because TIV is injected intramuscularly, some of the injected HA protein could be complexed by preexisting antibodies in circulation, which may reduce the amount of antigen available for stimulating naïve and/or memory B-cells. Atmar et al. found a significant positive correlation between dose of intramuscular administration of TIV and antibody titer after vaccination [44]. In contrast, replication of LAIV in the nasal mucosa is more directly related to mucosal immunity, including local cellular and humoral mediators, than to serum antibody [45]. Therefore, the magnitude of effector B-cell responses after ′05-LAIV may be less likely to be affected by preexisting circulating antibodies. It will be interesting to investigate the relationship between the B-cell responses and other cellular immune responses to LAIV compared to TIV in future studies.

In agreement with previous reports [35], [36], after vaccination with ′05-TIV, effector B-cells transiently appeared in the circulation with a sharp peak around day 7, and quickly disappeared thereafter (Figure 3 and [5]). The temporal appearance of circulating effector B-cells after LAIV immunization has not been studied previously. In the current study we observed a difference between LAIV and TIV in the kinetics of effector B-cell appearance in the circulation during the period from day 7 to 11 post-vaccination. The overall number of flu-specific ASC was significantly higher in the ′05-TIV recipients than the ′05-LAIV recipients (Figures 2 and 3), while the decline of both IgG and IgA effector B-cells was significantly faster in TIV recipients. The different kinetics are likely to reflect the mechanism of induction of immunity by the two vaccines. After an injection of TIV, naive or memory B-cells are exposed to a synchronized administration of non-replicating antigen, start to differentiate into effector B-cells, enter the circulation approximately 7 days later, and then traffic to the effector sites and disappear from circulation quickly. Induction of effector B-cells after intranasal delivery of LAIV is related to the asynchronous replication of LAIV, which is subjected to inter-personal variation in terms of actual dosing, antigen presentation and viral replication. Collectively these factors are likely to result in less synchronized B-cell responses among study subjects. Of note, our assay was not designed to measure the ASCs specific for individual influenza strains. Several vaccine studies showed that H3N2 was more immunogenic than H1N1 or B strains, and that TIV vaccination induced stronger antibody responses to all three strains while LAIV tended to induce weaker responses to H1N1 and B strains [5], [41]. Therefore, it is possible that the effector B-cells detected after TIV were specific for three different virus-specific ASC while those detected after LAIV were dominantly against H3N2. Further studies will be needed to examine the effector B-cell response against individual virus strains after these two types of vaccines.

The positive correlation between effector B-cell response and serum response (fold-increase of HAI antibody) after vaccination suggests that the serum response is associated with the effector B-cell response in the circulation. Although the HAI assay could not distinguish the subclass of antibody, IgG antibody is a generally dominant subclass in the serum and is a major contributor to HA inhibition. Serum IgG and local IgA levels are known to be correlated with protection from influenza infection [12]. Therefore the effector IgG ASC response could serve as a surrogate marker for protection efficacy of influenza vaccination. Although LAIV induces a higher mucosal antibody response than TIV [15], [16], we observed significantly higher numbers of effector IgA cells after TIV vaccination compared to LAIV (Figure 2 and [5]). Further analysis of the mucosal IgA response and its relationship to the effector IgA B-cell response in blood may clarify the relationship of circulating effector IgA B-cells to different types of vaccines. In addition, analysis for the expression of homing markers on the circulating effector B-cells and the expression of homing ligands at the site of immunization could help clarify the mechanism of induction of the local immune response after immunization with different influenza vaccines.

We observed a correlation between the baseline HAI titer and effector B-cell responses, but not between the baseline HAI levels and memory B-cell responses after vaccination, with the exception of an inverse correlation between HAI for H3N2 and memory IgA B-cell response in ′05-LAIV (Table 1). Therefore, the mechanisms for induction of effector B-cells might differ from those of memory B-cells, which were less influenced or not influenced by preexisting antibodies. Of note, the memory IgA B-cell increase was not statistically significant in ′05-LAIV group, indeed 63% of adults did not have increased memory IgA B-cell in ′05-LAIV (Figure 4). Interestingly, the adults whose memory IgA B-cell increased after ′05-LAIV had lower HAI titers against H3N2 before vaccination (6 out of 7 adults had HAI≤4). The remaining adults whose memory IgA B-cell did not increase in ′05-LAIV had preexisting HAI titer (HAI≥8, data not shown). The current study demonstrated an increase in memory B-cell response after both ′05-LAIV and ′05-TIV (Figure 4), while in our previous study of 2004 such an increase was not seen in LAIV recipients [5]. The different results between the 2004 and 2005 studies may also indicate an effect of prior year vaccination status on the immune responses after recent influenza vaccination. Amanna et al. studied antibody titer and memory B-cell numbers in the blood and found that there was no significant correlation between peripheral memory B-cell numbers and antibody levels for five of the eight antigens tested [46]. Further analysis of single strain-specific antibody and memory B-cell responses will be important for understanding the relationship between influenza-specific antibody and memory B-cell responses to LAIV and TIV, and their impact on protective immunity against influenza infection.

Based on our results, the effector B-cell response measured around day 7 could be used as a highly effective surrogate marker for vaccine “take” compared to the more traditional 30 day antibody response rate after LAIV and TIV vaccination. Earlier and more sensitive confirmation of vaccine “take” rates by surrogate markers would be especially useful in the case of LAIV vaccination where traditional serology assays seem to be very insensitive.

## References

[1] Thompson WW, Shay DK, Weintraub E, Brammer L, Bridges CB (2004). Influenza-Associated Hospitalizations in the United States.. JAMA.

[2] CDC CfDCaP (2005). Update: Influenza activity–United States and worldwide, 2004–05 season.. MMWR Morb Mortal Wkly Rep.

[3] CDC CfDCaP (2004). Update: influenza activity–United States and worldwide, 2003–04 season, and composition of the 2004–05 influenza vaccine.. MMWR Morb Mortal Wkly Rep.

[4] Nichol KL, Mallon KP, Mendelman PM (2003). Cost benefit of influenza vaccination in healthy, working adults: an economic analysis based on the results of a clinical trial of trivalent live attenuated influenza virus vaccine.. Vaccine.

[5] Sasaki S, Jaimes MC, Holmes TH, Dekker CL, Mahmood K (2007). Comparison of the Influenza Virus-Specific Effector and Memory B-Cell Responses to Immunization of Children and Adults with Live Attenuated or Inactivated Influenza Virus Vaccines.. J Virol.

[6] Zeman AM, Holmes TH, Stamatis S, Tu W, He XS (2007). Humoral and cellular immune responses in children given annual immunization with trivalent inactivated influenza vaccine.. Pediatr Infect Dis J.

[7] Plotkin JB, Dushoff J, Levin SA (2002). Hemagglutinin sequence clusters and the antigenic evolution of influenza A virus.. Proceedings of the National Academy of Sciences.

[8] McDonald NJ, Smith CB, Cox NJ (2007). Antigenic drift in the evolution of H1N1 influenza A viruses resulting from deletion of a single amino acid in the haemagglutinin gene.. J Gen Virol.

[9] Krystal M, Young JF, Palese P, Wilson IA, Skehel JJ (1983). Sequential Mutations in Hemagglutinins of Influenza B Virus Isolates: Definition of Antigenic Domains.. Proceedings of the National Academy of Sciences.

[10] Couch RB (1975). Assessment of immunity to influenza using artifical challenge of normal volunteers with influenza virus.. Dev Biol Stand.

[11] Couch RB, Kasel JA (1983). Immunity to influenza in man.. Annu Rev Microbiol.

[12] Clements ML, Betts RF, Tierney EL, Murphy BR (1986). Serum and nasal wash antibodies associated with resistance to experimental challenge with influenza A wild-type virus.. J Clin Microbiol.

[13] Belshe R, Gruber W, Mendelman P, Mehta H, Mahmood K (2000). Correlates of Immune Protection Induced by Live, Attenuated, Cold-adapted, Trivalent, Intranasal Influenza Virus Vaccine.. The Journal of Infectious Diseases.

[14] Belshe RB, Edwards KM, Vesikari T, Black SV, Walker RE (2007). Live Attenuated versus Inactivated Influenza Vaccine in Infants and Young Children.. N Engl J Med.

[15] Philip R, Johnson, SFJMTJDMPFW (1985). Comparison of long-term systemic and secretory antibody responses in children given live, attenuated, or inactivated influenza A vaccine.. Journal of Medical Virology.

[16] Moldoveanu Z, Clements ML, Prince SJ, Murphy BR, Mestecky J (1995). Human immune responses to influenza virus vaccines administered by systemic or mucosal routes.. Vaccine.

[17] Treanor JJ, Kotloff K, Betts RF, Belshe R, Newman F (1999). Evaluation of trivalent, live, cold-adapted (CAIV-T) and inactivated (TIV) influenza vaccines in prevention of virus infection and illness following challenge of adults with wild-type influenza A (H1N1), A (H3N2), and B viruses.. Vaccine.

[18] Nichol KL, Mendelman PM, Mallon KP, Jackson LA, Gorse GJ (1999). Effectiveness of Live, Attenuated Intranasal Influenza Virus Vaccine in Healthy, Working Adults: A Randomized Controlled Trial.. JAMA.

[19] Belshe RB, Mendelman PM, Treanor J, King J, Gruber WC (1998). The Efficacy of Live Attenuated, Cold-Adapted, Trivalent, Intranasal Influenzavirus Vaccine in Children.. N Engl J Med.

[20] Fiore AE, Shay DK, Haber P, Iskander JK, Uyeki TM (2007). Prevention and control of influenza. Recommendations of the Advisory Committee on Immunization Practices (ACIP), 2007.. MMWR Recomm Rep.

[21] Gross PA, Sperber SJ, Donabedian A, Dran S, Morchel G (1999). Paradoxical response to a novel influenza virus vaccine strain: the effect of prior immunization.. Vaccine.

[22] Keitel WA, Cate TR, Couch RB, Huggins LL, Hess KR (1997). Efficacy of repeated annual immunization with inactivated influenza virus vaccines over a five year period.. Vaccine.

[23] Smith DJ, Forrest S, Ackley DH, Perelson AS (1999). Variable efficacy of repeated annual influenza vaccination.. Proc Natl Acad Sci U S A.

[24] Hoskins TW, Davies J, Smith AJ, Miller C, Allchin A (1979). Assessment of inactivated influenza-A vaccine after three outbreaks of influenza A at Christ's Hospital.. The Lancet.

[25] Gross PA, Denning CR, Gaerlan PF, Bonelli J, Bernius M (1996). Annual influenza vaccination: immune response in patients over 10 years.. Vaccine.

[26] Gupta V, Earl DJ, Deem MW (2006). Quantifying influenza vaccine efficacy and antigenic distance.. Vaccine.

[27] Noble GR, Kaye HS, O'Brien RJ, Kendal AP, Bregman DJ (1977). Persistence of influenza A/New Jersey/76 (Hsw1N1) antibody one year after vaccination.. Dev Biol Stand.

[28] Clements ML, Murphy BR (1986). Development and persistence of local and systemic antibody responses in adults given live attenuated or inactivated influenza A virus vaccine.. J Clin Microbiol.

[29] Sheth KJ, Sedmak GV, Freeman ME, Eisenberg C (1979). Hemagglutination-inhibiting antibodies in vaccinated children with renal disease.. Jama.

[30] Kunzel W, Glathe H, Engelmann H, Van Hoecke C (1996). Kinetics of humoral antibody response to trivalent inactivated split influenza vaccine in subjects previously vaccinated or vaccinated for the first time.. Vaccine.

[31] Crotty S, Aubert RD, Glidewell J, Ahmed R (2004). Tracking human antigen-specific memory B cells: a sensitive and generalized ELISPOT system.. J Immunol Methods.

[32] Center for Disease Control and Prevention (1998). The 1998–99 WHO influenza reagent kit for the indentification of influenza isolates..

[33] Liang K-Y, Zeger SL (1986). Longitudinal data analysis using generalized linear models.. Biometrika.

[34] Holm S (1979). A simple sequentially rejective multiple test procedure.. Scandinavian Journal of Statistics.

[35] Cox RJ, Brokstad KA, Zuckerman MA, Wood JM, Haaheim LR (1994). An early humoral immune response in peripheral blood following parenteral inactivated influenza vaccination.. Vaccine.

[36] el-Madhun AS, Cox RJ, Soreide A, Olofsson J, Haaheim LR (1998). Systemic and mucosal immune responses in young children and adults after parenteral influenza vaccination.. J Infect Dis.

[37] Beyer WE, Palache AM, Luchters G, Nauta J, Osterhaus AD (2004). Seroprotection rate, mean fold increase, seroconversion rate: which parameter adequately expresses seroresponse to influenza vaccination?. Virus Res.

[38] Beyer WEP, Palache AM, de Jong JC, Osterhaus ADME (2002). Cold-adapted live influenza vaccine versus inactivated vaccine: systemic vaccine reactions, local and systemic antibody response, and vaccine efficacy: A meta-analysis.. Vaccine.

[39] Beyer WEP, Palache AM, Sprenger MJW, Hendriksen E, Tukker JJ (1996). Effects of repeated annual influenza vaccination on vaccine sero-response in young and elderly adults.. Vaccine.

[40] Lee MS, Mahmood K, Adhikary L, August MJ, Cordova J (2004). Measuring antibody responses to a live attenuated influenza vaccine in children.. Pediatr Infect Dis J.

[41] Ohmit SE, Victor JC, Rotthoff JR, Teich ER, Truscon RK (2006). Prevention of antigenically drifted influenza by inactivated and live attenuated vaccines.. N Engl J Med.

[42] Iorio AM, Neri M, Lepri E, Camilloni B, Basileo M (2006). An influenza A/H3 outbreak during the 2004/2005 winter in elderly vaccinated people living in a nursing home.. Vaccine.

[43] Nolan T, Lee MS, Cordova JM, Cho I, Walker RE (2003). Safety and immunogenicity of a live-attenuated influenza vaccine blended and filled at two manufacturing facilities.. Vaccine.

[44] Atmar RL, Keitel WA, Cate TR, Munoz FM, Ruben F (2007). A dose-response evaluation of inactivated influenza vaccine given intranasally and intramuscularly to healthy young adults.. Vaccine.

[45] Clements ML, O'Donnell S, Levine MM, Chanock RM, Murphy BR (1983). Dose response of A/Alaska/6/77 (H3N2) cold-adapted reassortant vaccine virus in adult volunteers: role of local antibody in resistance to infection with vaccine virus.. Infect Immun.

[46] Amanna IJ, Carlson NE, Slifka MK (2007). Duration of humoral immunity to common viral and vaccine antigens.. N Engl J Med.

